# Influence of vitamin D on muscle strength and botulinum toxin dosage through surface electromyography

**DOI:** 10.1590/acb396824

**Published:** 2024-10-07

**Authors:** Marcelle Simões Coelho, Gabriel Cirone Lopes, Luigi Giovanni Bernardo Sichi, Sigmar de Mello Rode, Rodrigo Máximo de Araújo

**Affiliations:** 1 Universidade Estadual Paulista “Júlio de Mesquita Filho” – Dental Materials and Prosthesis Departament – São José dos Campos (SP) – Brazil.

**Keywords:** Botulinum Toxins, Vitamin D, Self Concept, Muscle Strength, Skin Aging

## Abstract

**Purpose:**

To evaluate the influence of patients’ serum vitamin D levels on muscle strength characteristics and whether it impacts the durability of botulinum toxin (BT) treatment.

**Methods:**

The muscle strength of the frontal and corrugator muscles was evaluated before and after the application of TB with pre- and post-application control measurements, and at weeks 2, 5 and 12. The effect of vitamin D on muscle strength and its interaction with BT were investigated in 20 patients. The muscle contraction force was measured by surface electromyography.

**Results:**

The results revealed statistically significant differences between the frontal measurement groups at weeks 2 and 5, as well as for the corrugator in the same weeks and at week 12. Regarding vitamin D, significant differences were observed only in the initial group with vitamin D > 30 ng/mL compared to < 30 ng/mL for the frontal muscles. Patients with higher levels of vitamin D had higher average muscle strength compared to those with lower levels in all evaluations.

**Conclusions:**

It was observed that vitamin D influences muscle strength and the necessary dosage of BT.

## Introduction

We live in a time when quality of life is essential. Everyone wants to maintain a young appearance and continue having it as long as possible for their personal well-being and for professional needs, since the labor market excludes the elderly at an increasingly early age^1^. Due to the considerable increase in average life expectancy, there are an increasing number of elderly people, and yet, paradoxically, any visible sign of aging is mercilessly rejected by society. This growing demand for cosmetic treatments to reduce wrinkles and the sagging of the face has stimulated the literature’s search for non-surgical techniques to enhance facial beauty. Advancement in the use of toxins is an emerging science for facial beautification^2^.

Botulinum toxin (BT) inhibits the release of acetylcholine at the neuromuscular junction. The injection of small amounts of BT into specific hyperactive muscles causes localized muscle relaxation that softens the underlying skin and reduces wrinkles^3^. These wrinkles are triggered by the loss of collagen and muscle activity. In this way, the degradation is accelerated throughout aging, and this generates a disorganization and fragmentation mainly of the fibers of types I and III^4^. Its formation has two stages, the first of which is when it appears with movement, known as *dynamic wrinkle*, and the second one is when it is visible without the need for facial expression, known as *static wrinkle* 5. It is possible to visually verify the efficiency in the temporary disappearance of dynamic wrinkles after the use of BT.

The dosage and placement of the injection are based on the analysis of the target muscles and the adjacent muscles, in addition to the associated soft and hard tissues. The indication for BT selection as a primary intervention is excessive muscle contraction^6^.

The partial reduction in muscle function is seen from the third day after the application of BT, with a maximum visible reduction two weeks after application^5^. The typical duration of the effect of BT is three or four months, depending on several factors, including the dose, concentration, injection technique, patient’s immune response, and other factors^7^.

Bischoff-Ferrari et al.^8^ demonstrated that there is a correlation between vitamin D sufficiency and ideal muscle function. Increased vitamin D levels can reduce inflammation, pain, and myopathy while increasing muscle protein synthesis, adenosine triphosphate (ATP) concentration, and strength^6^^,^ 9. After activation to 1.25 hydroxyvitamin D3 -1.25 (OH) D, or calcitriol-vitamin D, the expression of the responsive gene is altered, with more than 1,000 vitamin D-responsive genes identified. These genes affect muscle protein synthesis, muscle strength, muscle size, reaction time, balance, coordination, endurance, inflammation, and immunity^9^.

The accepted definition of vitamin D sufficiency is 25-hydroxyvitamin D3 (25 [OH] D) between 20 and 30 ng/mL. However, it has been reported that more than one billion people worldwide are deficient in vitamin D, with 36 to 69.9% of children and adults suffering from this deficiency^10^. In addition, the rates of this deficiency doubled from 1994 to 2004, with several responsible factors, including avoidance of the sun, use of sunscreen, and increasing obesity rates causing the sequestration of this fat-soluble vitamin^11^.

A method that has been used to evaluate neuromuscular responses in various activities of healthy individuals and to research possible changes due to pathology or during certain types of muscle rehabilitation is electromyography (EMG). The EMG signal represents the characteristics of muscle function and provides information on muscle activities. Analyzing this signal can provide health professionals with diagnostic information that can serve as a useful tool when deciding on an appropriate treatment plan^12^. All these findings, combined with clinical evaluation, can provide useful evidence to assist patients over time, plan a specific rehabilitation treatment, and obtain the best performance for each area of activity^12^.

## Methods

This work evaluated the neuromuscular responses, through EMG, during expressive movements before and after the application of BT (Botulim 100U, Hugel, Inc., Chuncheon, South Korea). Five measurements were performed on each patient using the EMG:

- T1: pre-application measurement of BT;
- T2: measurement soon after application;
- T3: measurement 14 days after the application of BT;
- T4: measurement five weeks after the application of BT;
- T5: measurement 12 weeks after application of BT.

Each muscle had its strength measured three times on each side, in each take, resulting in six root mean square (RMS), effective value, values for each muscle group, which were frontal and corrugated, to generate greater data reliability. Each patient underwent a vitamin D test using chemiluminescence to check whether there was an influence of this vitamin on the muscular response.

According to the sample calculation, 20 patients were selected, with a sample power of 80%, which we evaluated using the vitamin D index (considered to be low level when below 30 ng/mL and the reference value when above 30 ng/mL). All patients who were eligible for the study signed a free and informed consent form.

The patients included in this study had dynamic wrinkles in the upper third of the face, body mass index between 18 and 35, in order to avoid possible influence on the storage of vitamin D, on adipocytes, in the body and who have not undergone the application of BT for less than six months.

Electromyography was performed through an eight-channel EMG-800C-832 electromyograph (EMG System do Brasil Ltda, São José dos Campos, SP, Brazil), previously calibrated, common mode rejection > 100dB, 16-bit analog-to-digital (A/D) converter board with dynamic band resolution, communication to the microcomputer using an Ethernet network adapter 10Mbits RJ45 (10BASE T) connector using TCP/IP protocol; double-pole Butterworth analog filter, low-pass (FPB) of 500 Hz and high-pass (FPA) of 20 Hz; software for the acquisition and analysis of electromyographic signals Windows Vista/XP platform for simultaneous presentation of the signals of multi-channels and Signal treatment (RMS value, mean, minimum, maximum, and standard deviation), FFT (online) with acquisition rate (sampling) of up to 2,000 samples/second per software programmable channel. The surface electrodes used were the Ag/AgCl bipolar, circular shape, disposable (Chicopee MA01, Meditrace^®^ Kendall-LTP, Dublin, Ireland), for simultaneous capture of the electrical activity of several motor units, providing a general approach to the muscle dynamics. These electrodes were coupled to a preamplifier with a 20-fold gain characterizing a differential circuit.

The dilution that was performed followed the pattern of 2 mL of serum for every 100 units of BT (Botulim 100U, Hugel, Inc., Chuncheon, South Korea). The application of this solution at each point was performed intramuscularly with the aid of a 1-mL syringe and a 30G needle.

In this way, it was possible to quantitatively evaluate the measurements by EMG to ascertain whether there was interference by this vitamin on the durability and effectiveness of BT.

The contraction results in RMS were analyzed according to the averages of each muscle. It was separated according to the muscle group analyzed, vitamin D (> or <), and time of measurement (initial, immediate, two, five, and 12 weeks). The mean values underwent statistical analysis using the Shapiro-Wilk’s test, Friedman’s test and Mann–Whitney’s U test for independent samples (α = 0.05).

To better elucidate the results obtained, it was proposed to evaluate the conservation of muscle strength over time, at two, five, and 12 weeks among individuals with vitamin D (> or <). For this, Eq. 1 was used:


$$\text{Muscle strenght}=\left(100^{*}MS\right)/MI$$ (1)

where: MS: the average of the week (2nd, 5th, or 12th); MI: the initial average.

The values of conservation of muscle strength over time were analyzed using the Shapiro-Wilk’s test (α = 0.05). For the frontal muscle group, we opted for the Student’s t-test (α = 0.05), and for the corrugator muscle group, we used the Mann–Whitney’s U test for independent samples (α = 0.05).

## Results

The RMS data of the electromyographic measurements performed in the patients passed the normality test, using the Shapiro-Wilk’s test, which found that not all groups followed normality.

To identify if there was a statistically significant difference and, if so, where it was present, the Durbin–Conover’s multiple comparison test was performed for each group of muscles. The results of the frontal are shown in Table 1, and the results of the corrugators are presented in Table 2.

**Table 1:** Multiple comparison test, Durbin–Conover, frontal.

Friedman < 0.001		Statistics	*p*-value
Baseline – Frontal	Immediate postoperative – Frontal	1.624	0.108
Baseline – Frontal	2 weeks – Frontal	5.498	< 0.001
Baseline – Frontal	5 weeks – Frontal	3.874	< 0.001
Baseline – Frontal	12 weeks – Frontal	2.874	0.005
Immediate postoperative – Frontal	2 weeks – Frontal	7.122	< 0.001
Immediate postoperative – Frontal	5 weeks – Frontal	5.498	< 0.001
Immediate postoperative – Frontal	12 weeks – Frontal	4.498	< 0.001
2 weeks – Frontal	5 weeks – Frontal	1.624	0.108
2 weeks – Frontal	12 weeks – Frontal	2.624	0.010
5 weeks – Frontal	12 weeks – Frontal	1.000	0.320

Source: Elaborated by the author.

**Table 2:** Multiple comparison test, Durbin–Conover, corrugators.

Friedman < 0.001		Statistics	*p*-value
Baseline – Corrugator	Immediate postoperative – Corrugator	0.230	0.819
Baseline – Corrugator	2 weeks – Corrugator	3.560	< 0.001
Baseline – Corrugator	5 weeks – Corrugator	4.135	< 0.001
Baseline – Corrugator	12 weeks – Corrugator	4.594	< 0.001
Immediate postoperative – Corrugator	2 weeks – Corrugator	3.790	< 0.001
Immediate postoperative – Corrugator	5 weeks – Corrugator	4.364	< 0.001
Immediate postoperative – Corrugator	12 weeks – Corrugator	4.824	< 0.001
2 weeks – Corrugator	5 weeks – Corrugator	0.574	0.567
2 weeks – Corrugator	12 weeks – Corrugator	1.034	0.304
5 weeks – Corrugator	12 weeks – Corrugator	0.459	0.647

Source: Elaborated by the author.

Through the data obtained for the frontal muscle, as presented in Table 1, it was observed that there was a statistically significant difference between the initial moment in relation to two weeks and five weeks. Through the data obtained for the corrugator muscles, as presented in Table 2, it was observed that there was a statistically significant difference between the initial moment in relation to two, five, and 12 weeks.

The electromyographic RMS values of each muscle that was evaluated at this time were separated by a group of patients with vitamin D > 30 ng/mL and a group of patients with vitamin D < 30 ng/mL. They were analyzed according to all moments of measurement of muscle contraction. The groups took the Shapiro–Wilk’s test and showed no normal distribution. The non-parametric Mann–Whitney’s test was thus selected for the independent samples.

**Figure 1:**
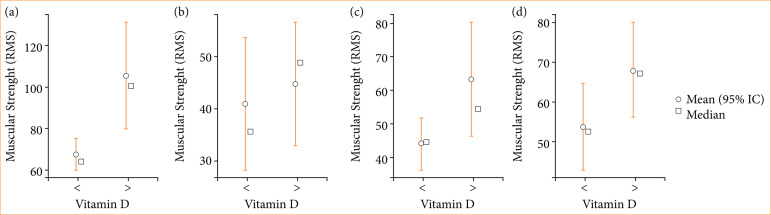
Average, median, minimum, and maximum of muscle strength: frontal muscle. **(a)** initial × vitamin D; **(b)** two weeks × vitamin D; **(c)** five weeks × vitamin D; **(d)** 12 weeks × vitamin D.

Source: Elaborated by the author.

In Table 3, it is observed that there was a statistically significant difference (*p* < 0.05) only for the groups of the initial frontal muscle with vitamin D > 30 ng/mL compared to vitamin D < 30 ng/mL. In Fig. 1a, the mean, median, minimum, and maximum of these groups are observed.

**Table 3:** Mann–Whitney’s U test for independent samples in root mean square, frontal and corrugators vitamin D < and > 30 ng/mL.

		Statistics	*p*-value
Baseline – Frontal	U Mann–Whitney	23.0	0.014
2 weeks – Frontal	U Mann–Whitney	51.0	0.582
5 weeks – Frontal	U Mann–Whitney	39.0	0.176
12 weeks – Frontal	U Mann–Whitney	35.0	0.107
Baseline – Corrugator	U Mann–Whitney	56.0	0.821
2 weeks – Corrugator	U Mann–Whitney	45.0	0.346
5 weeks – Corrugator	U Mann–Whitney	49.0	0.497
12 weeks – Corrugator	U Mann–Whitney	56.0	0.821

Source: Elaborated by the author.

The average obtained in the group of patients with vitamin D > 30 ng/mL was higher at all the patients’ follow-up times, as observed in Fig. 1. At the measurement times of two, five, and 12 weeks, there was no statistically significant difference between the groups.

**Figure 2:**
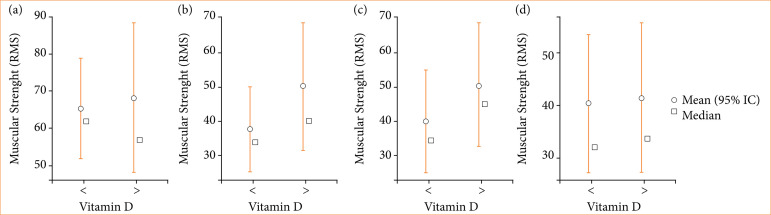
Mean, median, minimum, and maximum of muscle strength: corrugated muscle. **(a)** initial × vitamin D; **(b)** two weeks × vitamin D; **(c)** five weeks × vitamin D; **(d)** 12 weeks × vitamin D.

Source: Elaborated by the author.

In Table 3, it is observed that there was no statistically significant difference (p < 0.05) for any of the groups of the corrugator muscle. In Fig. 2, the mean, median, minimum, and maximum of all groups and moments is observed.

The average obtained in the group of patients with vitamin D > 30 ng/mL was higher than in the group of patients with vitamin D < 30 ng/mL at all follow-up times, as observed in Fig. 2.

Another possible way to evaluate muscle strength between the groups is through the percentage of the remaining strength after the application of BT (Table 4), no longer the RMS of muscle strength, between the groups of vitamin D > 30 ng/mL and vitamin D < 30 ng/mL. In this manner, the Shapiro-Wilk’s test was performed, and it was found that the frontal muscle data followed the normal distribution, thus enabling the realization of the t-test for independent samples. It was found that there was no statistically significant difference between any of the groups. For the groups of the corrugator muscle, the data did not follow the normal distribution. Consequently, the performance of the Mann–Whitney’s test proved to be adequate, and, through this test, it was found that there was no statistically significant difference between any of the groups.

**Table 4:** Descriptive statistics: percentages of remaining strength after botulinic toxin application, frontal and corrugators.

	Group	Mean	Median	Standard deviation
2 weeks – Frontal	<	64.2	56.6	37.0
	>	45.8	49.7	20.7
5 weeks – Frontal	<	68.6	70.2	26.7
	>	67.5	61.3	33.0
12 weeks – Frontal	<	87.5	83.2	22.3
	>	72.6	62.3	33.7
2 weeks – Corrugator	<	57.0	55.1	24.6
	>	75.5	70.4	31.8
5 weeks – Corrugator	<	59.4	55.3	35.4
	>	93.2	61.3	97.3
12 weeks – Corrugator	<	60.7	62.2	24.6
	>	76.4	43.3	82.7

Source: Elaborated by the author.

**Figure 3:**
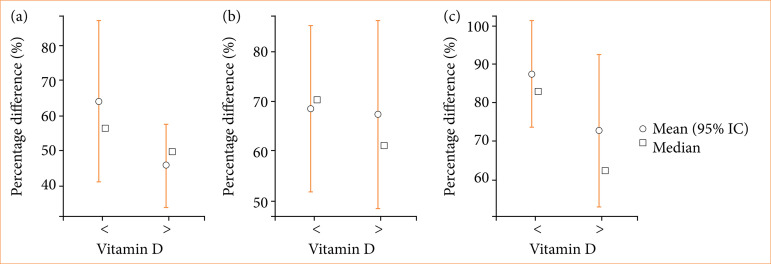
Average, median, minimum, and maximum of the percentage differences: frontal muscle. **(a)** initial × vitamin D; **(b)** two weeks × vitamin D; **(c)** five weeks × vitamin D; **(d)** 12 weeks × vitamin D.

Source: Elaborated by the author.

In Fig. 3, it is possible to observe the results of the frontal muscle. Even if there is no statistically significant difference in the absolute averages of the percentages, it is verified that the vitamin D group > 30 ng/mL had a greater decrease in strength compared to its own initial strength. This does not mean that they have a lower strength than the vitamin D group < 30 ng/mL in the absolute values of RMS.

Through the homogeneity test of Levene, variances were also observed; the data had a homogeneity of variances in addition to an absence of statistical differences.

**Figure 4:**
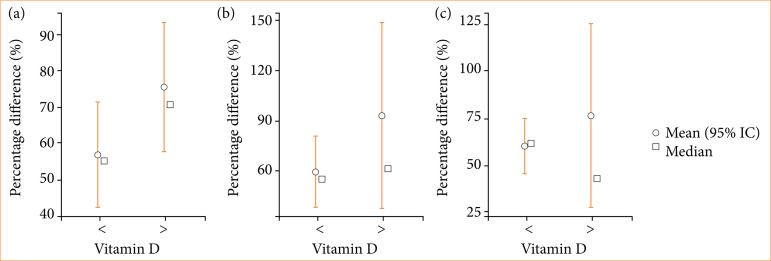
Average, median, minimum, and maximum graph of percentage differences: corrugated muscle. **(a)** initial × vitamin D; **(b)** two weeks × vitamin D; **(c)** five weeks × vitamin D; **(d)** 12 weeks × vitamin D.

Source: Elaborated by the author.

In Fig. 4, it is possible to observe the results of the corrugator muscle. Even if there is no statistically significant difference in the absolute averages of the percentages, it is verified that the vitamin D group < 30 ng/mL had a greater decrease in strength compared to its own initial strength, while the vitamin D group > 30 ng/mL presented a lower percentage of decrease in strength, which is the inverse of what occurred in the frontal muscle.

Through the homogeneity test of Levene, variances were also observed; the data had a homogeneity of variances in addition to an absence of statistical differences.

## Discussion

The BT dose that was used varied according to the extent and markings of the muscle to be treated. The greater the muscle strength or the more extensive the muscle, the more the wrinkles that were expressed and the higher the dose that needed to be used^13^. The average duration of the effect of BT in the upper part of the third of the face is between three and four months (as described in the bull Botulim® Blau Farmacêutica S/A), which may vary according to certain factors. These factors include the total dose, injection point, morphology of each muscle, topographic relationship with the surrounding muscles, and expressiveness of the patient. All these factors are relevant when optimizing the results^14^.

It was therefore not possible to perform a standard dosage for all patients in the study because, as mentioned, the dose needs to be individualized for each patient. The appropriate dosage was thus recommended to reduce the muscle strength of the region in an individualized way. An average of 36 IU per patient was used, thus obtaining improvements regarding wrinkles.

The selected reading times were determined so that it was possible to evaluate muscle strength before the intervention and at the time when the effect of BT was consolidated. This time was proven to be 14 days old^15^, because the BT that bound to the protein in the presynaptic cholinergic nerve inhibited the release of acetylcholine at the time in the region where it was applied. After the application of type A BT, it was found that the motor plate was fully restructured after 91 days. Additionally, effective muscle contraction may occur, which was observed in clinical practice^16^. The study was thus based on this period in order to determine the follow-up time for the patients, which was 12 weeks. According to these initial and final times of the BT effect already consolidated in the literature, an evaluation was added after a period of five weeks, so that it was possible to evaluate the strength during treatment.

EMG is a tool that allows an integrated study of the patient in the different clinical situations being analyzed. Although several studies have evaluated the effects of BT, few studies have been conducted to determine the long-term efficacy and durability of BT on the face, the monitoring of muscle strength, and the analysis of the vitamins possessed by each individual.

This lack of information about muscle activity possibly exists due to the limitations of the available registration techniques, which require physical restrictions or cable connections, thus restricting EMG studies. However, EMG brings us the measurements in RMS, which were reported to be highly reliable and reproducible indicators of muscle activity^17^, thus being selected for statistical analysis in this work.

In the clinical condition of rest, the electromyographic activity should be minimal or absent, with the muscles spontaneously relaxed^18^.

According to the data obtained in the study, the decrease in the muscle contraction strength of all patients was observed in relation to the initial measurement compared to at two weeks, at which time BT was at its greatest action potential in both muscles that were evaluated. It was thus possible to verify the effectiveness of BT in decreasing muscle strength.

In Table 1, it is observed that the moment of measurement immediately after application shows a small increase in strength. This is not statistically significant, but it may be associated with a protective contraction, which is an agonistic and synergistic association of the muscles to protect organs that are injured or threatened with damage. This occurs in the face with a pterygomandibular anesthesia, although there is a chance of injuring the medial pterygoid muscles, and the protective contraction reaction is the opening limitation.

The graphs that have been obtained through the EMG show high uniformity and consistency between the repeated exams, indicating that this type of study is repeatable and reliable. However, some patients may use one side of a muscle more often than the other side^19^, which does not change our results, since the average of the muscles was used for the statistical calculations of this study, as explained in the methodology.

EMG analysis is therefore useful in preoperative evaluation, postoperative follow-up, and the evaluation of results, which can facilitate the development of rehabilitation and physiotherapy programs^19^. It can also facilitate muscle analysis, assisting in aesthetic therapies and the evaluation of the systemic influence of the muscle groups being analyzed, as reported.

Vitamin D contributes to the development of muscle strength, as it acts on the formation of muscle fibers. It acts in the same way in relation to bones, as it is part of the basic and primordial process of the construction of tissues generated from the control of the supply of calcium and phosphorus to the body^20^. Therefore, given the importance of vitamin D in the construction of tissues, the same process is important for the contraction and proliferation of muscle fibers^21^.

The first associations between vitamin D and muscle function were made from observations of muscle weakness in children with rickets and in adults with osteomalacia^22^. The data obtained by Bischoff-Ferrari et al.^23^ suggested that the most advantageous target concentration of vitamin D starts at 75 nmol/L (30 ng/mL) and that the best concentrations are between 90 and 100 nmol/L (36–40 ng/mL). The groups selected for this study thus followed the recommendations of the Brazilian Society of Endocrinology and of several studies that indicate adequate levels of vitamin D. This resulted in the separation of patients into two groups: vitamin D > 30 ng/mL, and vitamin D < 30 ng/mL.

The statistical data obtained in this work thus demonstrated that the muscle strength of the patients in the vitamin D group > 30 ng/mL prior to any intervention corroborates the findings of the previously cited authors.

Therefore, according to the individualization of the BT dose and the records made in this study, an average dose of 40 iu was used for the group of patients with vitamin D > 30 ng/mL, and an average dose of 35 iu was used for the group of patients with vitamin D < 30 ng/mL. For the first time, this demonstrated the necessary difference of BT between patients with adequate serum levels and with vitamin D deficiency.

In Fig. 1, it is possible to observe the decrease in muscle strength in the frontal muscle after treatment and to observe a small increase in the average strength over the evaluation periods. These findings were not statistically relevant, but they demonstrate the beginning of muscle synaptic reinnervation, corroborating with the findings of Paiva et al.^16^.

In the analysis of the data regarding percentage differences in the frontal muscle, even though there was no statistically significant difference, it was possible to observe that the loss of strength was much more significant for patients with vitamin D > 30 ng/mL. This leads us to believe that the application of BT levels off the muscle activity of both groups to a more similar means of contraction strength. As the strength is much greater before the application in patients with vitamin D > 30 ng/mL, the loss is consequently also greater. This can be observed in Figs. 3 and 4.

## Conclusion

The results obtained showed that the dosage of vitamin D can have an influence by increasing the muscle strength of individuals in the region being studied. This can be validated through surface electromyography and by the need for a higher dosage of BT in patients with vitamin D > 30 ng/mL to present satisfactory results, as in the antagonist group. However, in relation to the durability of the treatment effects, no abbreviation of the effects was observed in any of the groups, even in those requiring a slightly higher dosage, and the duration in both groups was satisfactory.

Given the results presented and discussed in this work, it was concluded that the application of BT is effective for the aesthetic treatment in the upper third of the face, evaluated by muscle strength through electromyography.

## Data Availability

All data sets were generated or analyzed in the current study.
